# Trophic Factors and Cytokines in Early Diabetic Glomerulopathy

**DOI:** 10.1155/EDR.2003.225

**Published:** 2003

**Authors:** Frank C. Brosius III

**Affiliations:** 1 University of Michigan 1560 MSRB2 1150 W. Medical Center Drive Ann Arbor MI 48109-0676 USA; 2 Departments of Internal Medicine and Physiology University of Michigan Ann Arbor Michigan USA

## Abstract

The intent of this review is to focus on recent advances in the understanding of the factors responsible for the progressive pathologic features of diabetic kidney disease, with special attention to various growth factors and cytokines that appear to be important in this process. In addition, emphasis is centered on relatively early stages of the disease, because animal models have been most helpful to date in understanding this stage of the disease process. Although tubulointerstitial changes are of critical importance in the progression of diabetic nephropathy, especially in the evolution to end-stage renal disease, there is a general consensus that glomerular pathology occurs first. Therefore, attention is limited to factors that may be important in the development of early diabetic glomerulopathy, including transforming growth factor-beta (TGF-*β*), insulin-like growth factor (IGF)-I, vascular endothelial growth factor (VEGF)-A, and connective tissue growth factor (CTGF).


*Experimental Diab. Res.*, 4:225–233, 2003
Copyright © Taylor and Francis Inc.
ISSN: 1543-8600 print / 1543-8619 online
DOI: 10.1080/15438600390249682

Taylor & Francis
Taylor & Francis Group

### Trophic Factors and Cytokines in Early Diabetic Glomerulopathy

**Frank C. Brosius, III**

*Departments of Internal Medicine and Physiology, University of Michigan, Ann Arbor, Michigan, USA*

**The intent of this review is to focus on recent advances in the understanding of the factors responsible for the progressive pathologic features of diabetic kidney disease, with special attention to various growth factors and cytokines that appear to be important in this process. In addition, emphasis is centered on relatively early stages of the disease, because animal models have been most helpful to date in understanding this stage of the disease process. Although tubulointerstitial changes are of critical importance in the progression of diabetic nephropathy, especially in the evolution to end-stage renal disease, there is a general consensus that glomerular pathology occurs first. Therefore, attention is limited to factors that may be important in the development of early diabetic glomerulopathy, including transforming growth factor-beta (TGF-$\beta$), insulin-like growth factor (IGF)-I, vascular endothelial growth factor (VEGF)-A, and connective tissue growth factor (CTGF).**

**Keywords** Diabetes Mellitus; Diabetic Nephropathy; Growth Factors

### INTRODUCTION

The pathogenesis of diabetic nephropathy remains one of the most important enigmas in clinical nephrology. Diabetes is the most common cause of renal failure in the United States and in many developed countries, accounting for more than 43% of all patients reaching end-stage renal disease in the United States between 1996 and 2000 (U.S. Renal Data Systems, 2002). Moreover, the incidence of both diabetes and diabetic nephropathy has increased at near epidemic proportions during the last decade (U.S. Renal Data Systems, 2002). Although several clinical interventions, including tight glycemic control, treatment of hypertension and use of angiotensin-converting enzyme inhibitors and angiotensin receptor blockers, have been shown to retard the progression of nephropathy in many diabetic patients, the numbers of patients progressing to end-stage renal disease continues to increase yearly (U.S. Renal Data Systems, 2002). Thus, an improved understanding of the pathogenesis of this critical complication is being sought by many investigators around the world. The intent of this review is to focus on recent advances in our understanding of the factors responsible for the progressive pathologic features of diabetic kidney disease, with special attention to various growth factors and cytokines that appear to be important in this process. In addition, emphasis will be centered on relatively early stages of the disease, because animal models have been most helpful to date in understanding this stage of the disease process. Although tubulointerstitial changes are of critical importance in the progression of diabetic nephropathy, especially in the evolution to end-stage renal disease, there is a general consensus that glomerular pathology occurs first. Therefore, we will limit our attention to factors that may be important in the development of early diabetic glomerulopathy, including transforming growth factor-beta (TGF-$\beta$), insulin-like growth factor (IGF)-I, vascular endothelial growth factor (VEGF)-A, and connective tissue growth factor (CTGF).

### PATHOLOGY OF EARLY DIABETIC GLOMERULOPATHY

Over the past 20 years, it has become clear that relatively subtle morphological changes in the renal glomerulus occur

Received 16 January 2003; accepted 25 March 2003.
This work was supported in part by a grant from the National Institutes of Health (U01 DK060994) and from the Juvenile Diabetes Research Foundation Center of Excellence at the University of Michigan.
Address correspondence to Frank C. Brosius, University of Michigan, 1560 MSRB2, 1150 W. Medical Center Drive, Ann Arbor, MI 48109-0676, USA. E-mail: fbrosius@umich.edu quite early during the evolution of diabetic nephropathy and presage development of the diffuse and nodular sclerosis that are the characteristic lesions of overt diabetic glomerulopathy. Many studies have emphasized the widening of the glomerular basement membrane and expansion of the extracellular matrix area in the mesangium as the sites of important early changes. Mauer and colleagues showed that expansion of the extracellular mesangial matrix best predicted ultimate progression to frank glomerular sclerosis (Mauer et al., 1984). Logically, therefore, many investigators have emphasized factors that contribute to increased mesangial matrix accumulation in their research into the pathogenesis of early diabetic nephropathy.

More recently, it has become clear that the glomerular epithelial cell, or podocyte, is also damaged early in diabetes (Meyer et al., 1999; Pagtalunan et al., 1997; Hoshi et al., 2002b). Podocytes are highly complex cells comprised in part of arborizing processes terminating in interdigitating foot processes that envelop the glomerular capillaries and whose major function appears to be to create the filtration barrier that keeps circulating macromolecules within the circulation and out of the glomerular filtrate. Several studies of human diabetic glomeruli have strongly suggested that damage to podocytes may be just as important, if not more so, than the changes that occur in mesangial cells and in the extracellular glomerular mesangium. For example, Meyer and colleagues have shown that loss of glomerular podocytes is an early event in diabetic nephropathy and is highly predictive of both progressive glomerular injury and long-term albumin excretion in diabetic patients (Meyer et al., 1999; Pagtalunan et al., 1997). Because the mature podocyte has limited capacity to divide and to be replaced after injury, this loss from glomeruli caused by diabetes may eventually lead to albuminuria, glomerular scarring (glomerulosclerosis), and, ultimately, end-stage renal disease.

### EXPERIMENTAL MODELS OF DIABETIC NEPHROPATHY

A number of animal models of diabetic kidney disease have been investigated over the past several decades. Although several of these models recapitulate many or most of the early pathologic features of diabetic nephropathy just described, none of them fully reproduce the disease process found in humans. The features lacking from all currently described models are profound albuminuria (10- to 100-fold normal values) and diffuse or nodular glomerular sclerosis, followed by progressive decline in glomerular filtration rate to end-stage renal disease. Although some models appear to have one or another of these features, no reported model recapitulates this program. Thus, until such a model exists, conclusions about causal and pathogenic factors are necessarily provisional. However, many animal models do reasonably faithfully repeat the early changes of diabetic nephropathy: glomerular hyperfiltration (i.e., increased glomerular filtration rate), glomerular hypertrophy, widened glomerular basement membrane, mesangial matrix expansion, and podocyte damage or loss. Therefore, it appears that the current models should be able to shed some light on the early changes of diabetic nephropathy in humans.

Parenthetically, because mouse models have recently become more frequently utilized in the study of diabetes and its complications, it has become clear that serum creatinine measurements, performed by picric acid methods, do not reliably predict glomerular filtration rate in mice when compared to inulin clearance or other reliable methods (Animal Models of Diabetic Complications Consortium, unpublished data; www.amdcc.org). This appears to be due to competing chromagens in mouse serum that artifactually inflate the measured creatinine level severalfold in mice with normal renal function (Animal Models of Diabetic Complications Consortium, unpublished data). Accurate measurements of serum creatinine that give creatinine clearances that closely approximate inulin clearances can be obtained with high-performance liquid chromatography (HPLC) measurements of serum creatinine. Thus, all reports of serum creatinine or creatinine clearances using the picric acid (Jaffe) method on serum samples need to be reinterpreted in light of the preceding discussion.

### TROPHIC FACTORS AND CYTOKINES IN EARLY DIABETIC RENAL DISEASE

Many factors have been implicated in the initiation of these early glomerular changes in diabetes. In this short review, it will be impossible to give justice to the scope of the research that has been published in this area. Rather, this survey will focus on those factors that, in the author's opinion, are most clearly pathogenically associated with diabetic kidney disease, with some attention as well to newer and provocative reports. The effects of these factors on mesangial cell and, when possible, podocyte responses will be emphasized. Although many of the signaling events have been initially elucidated via cell-culture experiments, only those pathways with substantial validation from experimental animal models of diabetes will be emphasized. A general overview of some of these processes is outlined in Figure 1.

### GLUCOSE, GLUCOSE TRANSPORTERS AND ADVANCED GLYCATION END PRODUCTS (AGEs)

Although not usually classified as such, it is wise to recall that glucose is the ultimate trophic factor in diabetic nephropathy.

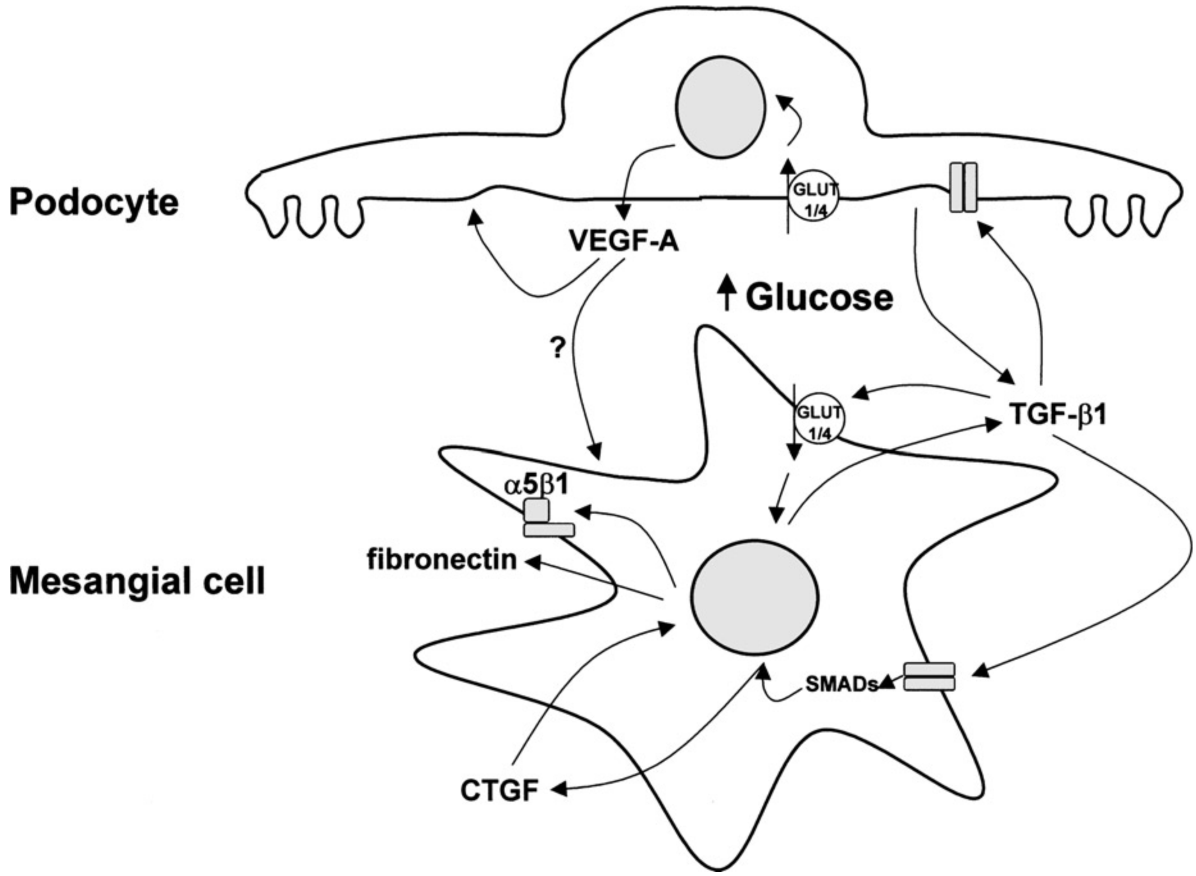

**FIGURE 1**

Growth factor effects in the diabetic glomerulus. High extracellular glucose is transported into both mesangial cells and podocytes by facilitative glucose transporters and engenders the elaboration of different growth factors from each of these cells. Several of these are represented in this figure with schematic representation of signaling and interactions. VEGF-A = vascular endothelial growth factor-A. TGF-$\beta 1$ = transforming growth factor-beta 1. CTGF = connective tissue growth factor. GLUT1/4 = facilitative glucose transporters 1 and 4. $\alpha 5\beta 1 = \alpha 5\beta 1$ integrin.

Similar to other factors, it interacts with a specific "receptor," in this case a facilitative glucose transporter, in order to enter a cell and have intracellular effects. Indeed, many cells are relatively protected from the effects of glucose because they have limited numbers of such transporters or reduce the plasma membrane expression of such transporters in the presence of elevated levels of extracellular glucose (Duelli et al., 2000; Hahn et al., 1998; Kaiser et al., 1993). This protective response appears to be lacking in renal glomerular mesangial cells in culture and in vivo, which, in fact, augment their levels of glucose transporters when extracellular glucose levels rise (Heilig et al., 1997b; D'Agord et al., 2001; Liu et al., 1999). Similar changes have been reported in retinal endothelial cells, another target tissue for complications in diabetes (Kumagai et al., 1996). The effect of high glucose levels on podocyte glucose transporters has not yet been reported. Many of the effects of elevated extracellular glucose can be reproduced simply by increasing the number of glucose transporters in cells and thereby enhancing glucose uptake and metabolic flux (Heilig et al., 1995, 1997b; Henry et al., 1999; Weigert et al., 2003). Conversely, many of the effects of elevated extracellular glucose can be prevented simply by reducing the number of plasma membrane transporters (Heilig et al., 2001).

The major glucose transporter in mesangial cells is the so-called constitutive transporter, GLUT1, although GLUT4, the insulin-responsive glucose transporter, is also expressed in these cells in culture and in vivo (Brosius et al., 1992). GLUT1 is the transporter that is expressed at increased levels in mesangial cells grown in high glucose (Heilig et al., 1997b) and in glomeruli in experimental animals (Brosius et al., unpublished observation). Although the glucose transporters in podocytes have not been well characterized, both GLUT1 and GLUT4 appear to be expressed (Brosius et al., 1992; Coward et al., 2002; Brosius et al., unpublished observation).

Glucose is the progenitor of another class of trophic factors that are often not regarded as such: AGEs. These adducts also have important signaling effects on glomerular cells. AGEs are generated through a series of nonenzymatic reactions between glucose and proteins or lipids and accumulate in diabetic patients at a much higher rate than they do in normals. High levels of AGEs have been implicated in the development of every diabetic complication. There is strong evidence that these end products contribute to the development of diabetic nephropathy and that inhibiting their formation with agents such as aminoguanidine can markedly ameliorate nephropathy in experimental animals (Vlassara and Palace, 2002; Abdel-Rahman and Bolton, 2002), though such trials have been less conclusive in human diabetic patients (Abdel-Rahman and Bolton, 2002). Although AGEs have extracellular effects, such as protein cross-linking, that appear to promote the expansion of the glomerular mesangial matrix and basement membrane in diabetic nephropathy, they also affect glomerular cells in a manner similar to growth factors and other ligands, by binding to a series of receptors known as RAGEs (receptor for advanced glycation end products). Ligation of these receptors leads to the stimulation of a number of cytokines, including TGF-$\beta$ (see below), as well as increased synthesis of extracellular matrix proteins. Thus, AGEs may be one of the proximal "growth factors" in driving the cellular machinery of the glomerulus to overproduce matrix proteins and engender other changes of diabetic kidney disease.

A full review of the effects of AGEs and extracellular glucose on diabetic glomeruli is beyond the scope of this article. The remaining discussion will focus on growth factors and cytokines as conventionally defined.

### TGF-$\beta$

TGF-$\beta$ has probably received more recent attention than any other trophic factor, in both the initiation and progression of diabetic nephropathy. There are three members of the TGF-$\beta$ family expressed in mammalian cells, TGF-$\beta$1, TGF-$\beta$2, and TGF-$\beta$3 (Cheng and Grande, 2002). Acting through two plasma membrane receptors, these peptide factors have myriad effects on cell growth and development, cell cycle regulation, inflammation and immune responses, extracellular matrix protein accumulation, and a number of other processes. These signals are generally transduced by transcription factors of the SMAD protein family. These factors lead to gene regulation events responsible for the profibrotic and hypertrophic effects of TGF-$\beta$ that have been implicated in a number of progressive renal diseases (Cheng and Grande, 2002; Flyvberg, 2000).

TGF-$\beta$1 and TGF-$\beta$1 receptors are expressed in glomerular cells, including mesangial cells and podocytes (MacKay et al. 1989; Ziyadeh et al., 1994; Abbate et al., 2002; Schiffer et al., 2001, 2002). Elevated extracellular glucose levels as well as exposure to glycosylated albumin lead to enhanced TGF-$\beta$ production as well as TGF-$\beta$ signal transduction in cultured mesangial cells (Ayo et al., 1990; Cohen et al., 1995). Similarly, experimental diabetes increases glomerular TGF-$\beta$1 production and signaling (D'Agord et al., 2001; Shankland and Scholey, 1994; Sharma and Ziyadeh 1994). Moreover, autocrine stimulation of mesangial cell TGF-$\beta$1 receptors by high glucose enhances extracellular matrix protein accumulation (Ziyadeh et al., 1994) and is associated with renal hypertrophy in animal models (Shankland and Scholey, 1994; Sharma and Ziyadeh, 1994). Finally, enhanced TGF-$\beta$ induces GLUT1 expression in mesangial cells (Inoki et al., 1999), leading to increased glucose metabolic flux and enhanced matrix production as noted above. Thus, TGF-$\beta$ action in glomerular mesangial cells leads to changes that appear to be central to the development of diabetic nephropathy.

Recent reports indicate that cultured podocytes respond somewhat differently than do mesangial cells in terms of TGF-$\beta$ signaling (Iglesias-de la Cruz et al., 2002). Elevated extracellular glucose levels do not increase TGF-$\beta$1 mRNA or protein in the podocyte, but elevated glucose did induce expression of the ligand-binding TGF-$\beta$ type II receptor, suggesting that enhanced TGF-$\beta$ signaling may ensue (Iglesias-de la Cruz et al., 2002). The role of TGF-$\beta$ signaling in the podocyte is just beginning to be elucidated. Whether such signaling is important in the development of diabetic glomerular changes remains to be determined.

Perhaps the most compelling data that implicate TGF-$\beta$ production and signaling in the pathogenesis of experimental diabetic nephropathy are studies by Ziyadeh and colleagues demonstrating the prevention or amelioration of diabetic nephropathy by administration of specific anti–TGF-$\beta$ antibodies. Reduction of TGF-$\beta$ signaling by administration of anti–TGF-$\beta$ antibodies starting at 8 weeks of age in db/db mice (approximately 4 weeks after onset of diabetes) prevented the increase in albuminuria and accumulation of mesangial matrix protein seen in mice treated with an unrelated antibody (Ziyadeh et al., 2000). More recent studies by the same group have shown that glomerular basement membrane (GBM) thickening and mesangial matrix expansion could be reversed by administration of these antibodies to db/db mice starting at 16 weeks of age (Chen et al., 2003).

Even though TGF-$\beta$1 levels increase early in diabetic nephropathy (Shankland and Scholey, 1994), it is not certain at what time point in this progression that its effects are most important. It is clear that certain features of diabetic nephropathy can occur without a significant increase in TGF-$\beta$ levels, at least in experiments in cultured cells. A recent report has found that exposure to high glucose increases protein kinase C (PKC) activity, PKC$\alpha$ levels, and fibronectin synthesis in cultured mesangial cells, despite a lack of change in TGF-$\beta$ levels. These recent data indicate that increased glucose uptake and metabolism induce PKC-dependent activator protein (AP)-1 activation that is sufficient for enhanced fibronectin production, but not for increased TGF-$\beta$1 expression (Weigert et al., 2003). Despite such data, TGF-$\beta$ appears to be a critical factor in the progression of diabetic nephropathy in experimental animals. Whether it is critical in the earliest changes that set the process in motion is somewhat less certain.

Finally, it should be mentioned that other members of the TGF-$\beta$ family may participate in the pathogenesis of diabetic nephropathy. However, there have been few investigations regarding these factors. One of these, bone morphogenetic factor-7 (BMP-7), has been found to decline in kidneys from streptozotocin diabetic rats (Wang et al., 2001). There is evidence that BMP-7 plays an antifibrotic role in kidney proximal tubules, and therefore decreased expression of this factor in diabetes may augment tubulointerstitial fibrosis. Whether it has effects on mesangial expansion or podocyte damage in early diabetic nephropathy has not been established.

### GROWTH HORMONE (GH) AND IGF-I

GH, IGF-I, and the GH/IGF-I axis have been implicated in the development of diabetic complications for some time. Although a number of studies support the role of these factors in the development of diabetic nephropathy, there remains less clear-cut evidence for their participation than for TGF-$\beta$. GH levels vary between models but tend to be elevated in several diabetic models (Flyvberg, 2000). GH induces expression of IGF-I in many tissues, including kidney (Flyvberg, 2000). In addition, renal levels of IGF-I are elevated in some (Flyvberg, 2000) but not all (Bach et al., 2000) reports of experimental models of diabetes. Even in models with increased IGF-I levels, there are increased levels of IGF-binding proteins (IGF-BPs), some of which may counteract the signaling effects of IGF-I. In addition, circulating IGF-I levels are depressed in diabetes (Thrailkill, 2000), so the changes in the GH/IGF-I axis in diabetic kidneys are quite complex.

The effects of GH and IGF-I on glomerular cells are also complex, although in general both promote glomerular hypertrophy and accumulation of extracellular mesangial matrix proteins (Baud et al., 1999). Most GH effects on glomerular hypertrophy appear to be IGF-I dependent, but at least in some cases GH effects on glomerular sclerosis may be independent of IGF-I (Baud et al., 1999). In accord with this, infusion of IGF-I appears to enhance renal hypertrophy in diabetic rats without albuminuria, whereas GH-deficient rats appear to have blunted glomerular hypertrophy as well as albuminuria when compared to control rats (Gronbaek et al.). In addition, IGF-I can also have direct effects on mesangial matrix protein accumulation. For example, IGF-I treatment of mesangial cells from nondiabetic NOD mice resulted in a decrease in metalloproteinase (MMP)-2 activity, whereas treatment with a neutralizing antibody against IGF-I restored the active form of MMP-2 (Lupia et al., 1999). MMP-2 is a proteinase that is critical in degrading extracellular matrix proteins, so IGF-I–induced inhibition of this metalloproteinase could lead to enhanced accumulation of extracellular matrix in diabetic glomeruli.

Results of studies with the GH antagonist, somatostatin, and its analogs are also supportive of the role IGF-I in augmenting diabetic nephropathy. Somatostatin inhibits GH and IGF-I action through a decrease in GH secretion and/or IGF-I expression or through a direct blockade of glomerular cell proliferation. Several groups have found that long-acting somatostatin analogs retard the development of mesangial expansion and albuminuria (Flyvberg, 2000). For example, a recent report showed that PTR-3173, a novel somatostatin analogue that has prolonged inhibitory action on the GH/IGF-I axis, counteracted some of the changes in kidney weight, glomerular volume, albuminuria, and creatinine clearance seen in untreated diabetic NOD mice (Landau et al., 2001). Another long-acting somatostatin compound, octreotide, was tested on renal function following induction of streptozotocin-diabetic rats. Chronic administration of octreotide for 7 days prevented the decrease of effective renal vascular resistance, and the increases in filtration fraction and glomerular filtration rate induced by diabetes. However, it only partly prevented the renal hypertrophy due to diabetes (Bak et al., 2001). Finally, IGF-I can induce GLUT1 expression in mesangial cells independent of the effects of extracellular glucose on GLUT1 levels (Heilig et al., 1997b). Therefore, IGF-I may augment the injury due to increased intracellular glucose in these cells. However, whether IGF-I promotes this effect in vivo has not yet been determined.

In sum, these studies generally support the conclusion that enhanced IGF-I signaling probably occurs in diabetic glomerular cells and that such signaling contributes to glomerular hypertrophy and possibly to accumulation of extracellular matrix proteins in early diabetic nephropathy. It seems unlikely that IGF-I or GH plays a central role in the progression of diabetic nephropathy because many of these events can be augmented by TGF-$\beta$ independent of IGF-I. Moreover, TGF-$\beta$ expression and action do not appear to be critically dependent on IGF-I signaling. Finally, somatostatin analogs, which have additional effects that are not due to changes in the GH/IGF-I axis, only partly inhibit the development of early diabetic nephropathy.

### VEGF-A

Recently, a number of studies have examined the role of VEGF-A in promoting diabetic nephropathy. In part this has been driven by the growing evidence that VEGF-A is a pathogenic factor in the evolution of diabetic retinopathy. In addition, it has recently become clear that VEGF-A has very important renal glomerular effects. VEGF-A mRNA and protein are expressed by mesangial cells (Iglesias-de la Cruz et al., 2002; Yamagishi et al., 2002) and podocytes (Hoshi et al. 2002b) in culture, but the most critical expression of VEGF-A in vivo in the kidney appears to be in the podocyte. A very recent report has shown that both over- and underexpression of the VEGF-A gene in the podocyte lead to significant glomerular abnormalities, including albuminuria and endotheliosis (underexpression) or collapsing glomerulopathy (overexpression) (Eremina et al., 2003). VEGF-A mRNA expression is induced in glomerular cells by high glucose or AGEs (Iglesias-de la Cruz et al., 2002; Yamagishi et al., 2002; Hoshi et al., 2002b) and is enhanced in renal cortex from obese diabetic Zucker fa/fa rats when compared to lean, nondiabetic control rats (Hoshi et al., 2002a).

In studies conceptually similar to those performed with TGF-$\beta$ antibodies, two groups investigated whether a neutralizing VEGF antibody would ameliorate changes of diabetic nephropathy in experimental diabetes, either streptozotocin-diabetic rat or db/db mouse models (de Vriese et al., 2001; Flyvberg et al., 2002). The animals received the antibody for 6 to 8 weeks. In the VEGF antibody–treated animals, kidney weight, glomerular hypertrophy, basement membrane thickness, hyperfiltration, and urinary albumin levels were all diminished compared to the diabetic groups receiving the irrelevant immunoglobulin G (IgG) injections. VEGF antibody administration tended to reduce expansion of total mesangial volume seen in diabetic animals, but this was not statistically significant. In one study, the investigators also noted that the increase in creatinine clearance (as an estimate of glomerular filtration rate) seen in the control diabetic animals was abolished by the VEGF antibody treatment (Flyvberg et al., 2002). However, serum creatinine levels were performed using the picric acid (Jaffe) method and therefore these values and the creatinine clearances that were calculated using them are suspect, as noted above. Thus, many but not all of the early features of diabetic nephropathy were ameliorated by chronic inhibition of VEGF in db/db mice.

Recent studies also suggest that elevated circulating VEGF levels is not a common feature of early diabetic kidney disease in humans, and in fact, those with early indications of progressive kidney disease may have lower levels of VEGF than those who have no markers of kidney disease (Shimada et al., 2002). Although kidney and glomerular levels may differ from those in the blood, these findings question the importance of VEGF as a pathogenic factor. Finally and critically, in some studies in nondiabetic humans and animals, infusion of neutralizing anti-VEGF antibodies or soluble VEGF receptors is associated with proteinuria (Sugimoto et al., 2003), suggesting that reduction of VEGF levels can lead to glomerular dysfunction and pathology. Thus, it remains unclear whether VEGF plays a pathogenic or protective role, or both, in glomeruli in diabetes. More definitive studies will be necessary before any firm conclusions can be drawn. What is certain, however, is that VEGF-A plays a critical role in maintaining glomerular integrity, so it will not be surprising to find that relatively subtle changes in VEGF-A levels or signaling lead to glomerular abnormalities in diabetic kidney disease.

### CTGF

During the past several years, there has been growing interest in CTGF (also known as CCN2) as an inducer of early diabetic nephropathy. It has been shown that this growth factor is induced in glomeruli from db/db diabetic mice after 3.5 months of diabetes (Riser et al., 2000) and in streptozotocin-diabetic rats after 32 weeks (Twigg et al., 2002). CTGF protein is also present in glomeruli of human patients with diabetic nephropathy (Wahab et al., 2001). Exposure of cultured mesangial cells to high glucose causes a twofold rise in CTGF protein levels within 48 hours (Riser et al., 2000). At least under these experimental conditions, CTGF appears to act downstream of TGF-$\beta$ (Riser et al., 2000; Wahab et al., 2001; Abdel-Wahab et al., 2002). AGEs also stimulate CTGF expression in cultured mesangial cells (Twigg et al., 2001, 2002). Treatment of streptozotocin-diabetic rats with aminoguanidine, an inhibitor of AGE formation, reduced CTGF to normal levels (Twigg et al., 2002). Inhibition of CTGF expression in mesangial cells by an antisense oligonucleotide approach or a chick anti-CTGF neutralizing antibody resulted in a diminution of high glucose–induced fibronectin accumulation in cultured mesangial cells (Wahab et al., 2001). Finally, it appears that both TGF-$\beta$ and CTGF act to induce fibronectin accumulation, in part via induction of $\alpha 5\beta$ integrin, a fibronectin receptor on mesangial cells (Weston et al., 2003). In this study, virtually all of TGF-$\beta$ effects were eliminated by CTGF antisense oligonucleotides.

A recent report has shown that CTGF stimulates cultured human mesangial cells to enter the G(1) phase from G(0), but then to arrest. CTGF was shown to stimulate expression of the cyclin-dependent kinase inhibitors (CDKIs) p15(INK4), p21(Cip1), and p27(Kip1), which inactivate cyclins D and E complexes, which could thereby inhibit cel-cycle progression. These findings imply that elevated CTGF levels may account for some of the mesangial cell hypertrophy seen in early diabetic nephropathy. Moreover, in this report, blockade of CTGF signaling interrupted TGF-$\beta$–induced hypertrophy, suggesting that CTGF acts downstream of TGF-$\beta$ in this system as well (Abdel-Wahab et al., 2002).

These recent data suggest that CTGF may serve as an important mediator of TGF-$\beta$–induced glomerular mesangial matrix accumulation in diabetic animals. Whether CTGF can be induced independently of TGF-$\beta$ or whether it is important in earlier changes of diabetic nephropathy remain to be elucidated. Nonetheless, its effects may be more specific than those of TGF-$\beta$ and thereby potentially more amenable to therapeutic intervention.

### OTHER CYTOKINES OR GROWTH FACTORS

Several studies have implicated platelet-derived growth factor (PDGF) and epidermal growth factor (EGF) in promoting diabetic nephropathy. PDGF mRNA levels are reported to be increased in streptozotocin-diabetic rats after 6 months of diabetes (Kelly et al., 2001). However, there is little evidence pointing to a direct role for PDGF in the evolution of early diabetic kidney disease. In fact, there appears to be no aggravating role of PDGF in diabetic condition because treatment with PDGF failed to lead to any development of progressive diabetic nephropathy in Goto Kakizaki diabetic rats, a genetic model of nonobese type 2 diabetes mellitus in which progressive diabetic kidney disease does not develop (Riley et al., 1999).

Similarly, several reports of increased EGF expression raised the possibility that this growth factor also participated in the development of diabetic nephropathy. Indeed, the kidney is one of the major sites of EGF production, though it is largely produced in cells in the distal nephron and unlikely to activate receptors that are known to exist in mesangial cells (Harris et al., 1988). As yet, there are no reports of EGF or EGF receptor expression in podocytes. Urinary EGF levels rise within the first week of diabetes but renal levels do not appear to be increased (Gilbert et al., 2002). There are no direct studies that have investigated the role of EGF signaling in promotion of the cardinal events of early diabetic nephropathy.

### SUMMARY

The changes of early diabetic nephropathy set the stage for progressive glomerulosclerosis and tubulointerstitial fibrosis and atrophy that characterize the failing diabetic kidney. A number of trophic factors and cytokines appear to promote these early changes by effects on both mesangial cells and podocytes. The precise nature of these nephropathy-promoting effects still remain to be elucidated and the manner in which the various factors interact to induce nephropathy has yet to be really studied. Nonetheless, these factors and cytokines, as well as the signaling events they trigger in mesangial cells and podocytes, are important potential targets for therapeutic efforts designed to forestall progressive diabetic nephropathy. Successful treatment of early diabetic nephropathy would have profound effects on the well-being and life expectancies of millions of diabetic patients.

### REFERENCES

Abbate, M., Zoja, C., Morigi, M., Rottoli, D., Angioletti, S., Tomasoni, S., Zanchi, C., Longaretti, L., Donadelli, R., and Remuzzi, G. (2002) Transforming growth factor-beta 1 is up-regulated by podocytes in response to excess intraglomerular passage of proteins: A central pathway in progressive glomerulosclerosis. *Am. J. Pathol.,* **161,** 2179–2193.

Abdel-Rahman, E., and Bolton, W. K. (2002) Pimagedine: A novel therapy for diabetic nephropathy. *Expert Opin. Investig. Drugs,* **11,** 565–574.

Abdel-Wahab, N., Weston, B. S., Roberts, T., and Mason, R. M. (2002) Connective tissue growth factor and regulation of the mesangial cell cycle: Role in cellular hypertrophy. *J. Am. Soc. Nephrol.,* **13,** 2437–2445.

Ayo, S. H., Radnik, R. A., Garoni, J., Glass, W. F., and Kreisberg, J. I. (1990) High glucose causes an increase in extracellular matrix proteins in cultured mesangial cells. *Am. J. Pathol,* **136,** 1339–1348.

Bach, L. A., Dean, R., Youssef, S., and Cooper, M. E. (2000) Aminoguanidine ameliorates changes in the IGF system in experimental diabetic nephropathy. *Nephrol. Dial. Transplant.,* **15,** 347–354.

Bak, M., Thomsen, K., and Flyvbjerg, A. (2001) Effects of the somatostatin analogue octreotide on renal function in conscious diabetic rats. *Nephrol. Dial. Transplant,* **16,** 2002–2007.

Baud, L., Fouqueray, B., Bellocq, A., Doublier, S., and Dumoulin, A. (1999) Growth hormone and somatostatin in glomerular injury. *J. Nephrol.,* **12,** 18–23.

Brosius, F. C., III, Briggs, J., Marcus, R., Barac-Nieto, M., and Charron, M. J. (1992) Expression of the insulin-responsive glucose transporter (GLUT4) in renal microvessels and glomeruli. *Kidney Int.,* **42,** 1086–1092.

Chen, S., Iglesias-de la Cruz, M. C., Jim, B., Hong, S. W., Isono, M., and Ziyadeh, F. N. (2003) Reversibility of established diabetic glomerulopathy by anti-TGF-$\beta$ antibodies in db/db mice. *Biochem. Biophys. Res. Commun.,* **300,** 16–22.

Cheng, J., and Grande, J. P. (2002) Transforming growth factor-$\beta$ signal transduction and progressive renal disease. *Exp. Biol. Med.,* **227,** 943–956.

Cohen, M. P., Hud, E., Wu, V. Y., and Ziyadeh, F. N. (1995) Albumin modified by Amadori glucose adducts activates mesangial cell type IV collage gene transcription. *Mol. Cell Biochem.,* **151,** 61–67.

Coward, R. J. M., Welsh, G. I., Koziell, A., Ni, L., Tavare, J. M., Mathieson, P. W., and Saleem, M. A. (2002) The podocyte is an insulin responsive, glucose utilizing cell; with a functional GLUT4/VAMP2 apparatus. An unexplored avenue in diabetic nephropathy? *J. Am. Soc. Nephrol.,* **13,** 45A.

D'Agord, S. B., Lacchini, S., Bertoluci, M. C., Irigoyen, M. C., Machado, U. F., and Schmid, H. (2001) Increased renal GLUT1 abundance and urinary TGF-beta 1 in streptozotocin-induced diabetic rats: Implications for the development of nephropathy complicating diabetes. *Horm. Metab. Res.,* **33,** 664–669.

de Vriese, A. S., Tilton, R. G., Elger, M., Stephan, C. C., Kriz, W., and Lameire, N. H. (2001) Antibodies against vascular endothelial growth factor improve early renal dysfunction in experimental diabetes. *J. Am. Soc. Nephrol.,* **12,** 993–1000.

Duelli, R., Maurer, M. H., Staudt, R., Heiland, S., Duembgen, L., and Kuschinsky, W. (2000) Increased cerebral glucose utilization and decreased glucose transporter Glut1 during chronic hyperglycemia in rat brain. *Brain Res.,* **858,** 338–347.

Eremina, V., Sood, M., Haigh, J., Nagy, A., Lajoie, G., Ferrara, N., Gerber, H. P., Kikkawa, Y., Miner, J. H., and Quaggin, S. E. (2003) Glomerular-specific alterations of VEGF-A expression lead to distinct congenital and acquired renal diseases. *J. Clin. Invest.,* **111,** 707–716.

Flyvbjerg, A. (2000) Putative pathophysiological role of growth factors and cytokines in experimental diabetic kidney disease. *Diabetologia,* **43,** 1205–1223.

Flyvbjerg, A., Dagnaes-Hansen, F., De Vriese, A. S., Schrijvers, B. F., Tilton, R. G., and Rasch, R. (2002) Amelioration of long-term renal changes in obese type 2 diabetic mice by a neutralizing vascular endothelial growth factor antibody. *Diabetes,* **51,** 3090–3094.

Gilbert, R. E., Cox, A., McNally, P. G., Wu, L. L., Dziadek, M., Cooper, M. E., and Jerums, G. (2002) Attenuation of tubular apoptosis by blockade of the renin-angiotensin system in diabetic Ren-2 rats. *Kidney Int.,* **61,** 31–39.

Gronbaek, H., Volmers, B. P., Bjorn, S. F., Osterby, R., Orskov, H., and Flyvbjerg, A. (1997) Effect of isolated GH and IGF-1 deficiency on long-term renal changes and urinary albumin excretion in streptozotocin diabetic dwarf rats. *Am. J. Physiol.,* **272,** E918–E924.

Hahn, T., Barth, S., Weiss, U., Mosgoeller, W., and Desoye, G. (1998) Sustained hyperglycemia in vitro down-regulates the GLUT1 glucose transport system of cultured human term placental trophoblast: A mechanism to protect fetal development? *FASEB J.,* **12,** 1221–1231.

Harris, R. C., Hoover, R. I., Jacobsen, H. F., and Badr, K. F. (1988) Evidence for glomerular actions of epidermal growth factor in the rat. *J. Clin. Invest.,* **82,** 1028–1039.

Heilig, C. W., Brosius, F. C., III, and Henry, D. N. (1997a) Glucose transporters of the glomerulus and the implications for diabetic nephropathy. *Kidney Int.,* **60,** S91–S99.

Heilig, C. W., Concepcion, L. A., Riser, B. L., Freytag, S. O., Zhu, M., and Cortes, P. (1995) Overexpression of glucose transporters in rat mesangial cells cultured in a normal glucose milieu mimics the diabetic phenotype. *J. Clin. Invest.,* **96,** 1802–1814.

Heilig, C. W., Kreisbergh, J. I., Freytag, S., Murakami, T., Ebina, Y., Guo, L., Heilig, K., Jin, Y., Henry, D., and Brosius, F. C., III. (2001) Antisense-GLUT1 protects mesangial cells from D-Glucose-induced transporter and fibronectin expression. *Am. J. Physiol.,* **280,** F657–F666.

Heilig, C. W., Liu, Y., England, R. L., Freytag, S. O., Gilbert, J., Zhu, M., Concepcion, L. A., and Brosius, F. C., III. (1997b) D-Glucose stimulates mesangial cell GLUT1 expression, and basal and IGF1-sensitive glucose uptake in rat mesangial cells: Implications for diabetic nephropathy. *Diabetes,* **46,** 1030–1039.

Henry, D. N., Buski, J. V., Concepcion, L. A., Brosius, F. C., III, and Heilig, C. W. (1999) Glucose transporters control the expression of aldose reductase, PKC$\alpha$, and GLUT1 genes in mesangial cells in vitro. *Am. J. Physiol.,* **277,** F97–F104.

Hoshi, S., Nomoto, K., Kuromitsu, J., Tomari, S., and Nagata, M. (2002a). High glucose induced VEGF expression via PKC and ERK in glomerular podocytes. *Biochem. Biophys. Res. Commun.,* **290,** 177–184.

Hoshi, S., Shu, Y., Yoshida, F., Inagaki, T., Sonoda, J., Watanabe, T., Nomoto, K., and Nagata, M. (2002b) Podocyte injury promotes progressive nephropathy in Zucker diabetic fatty rats. *Lab. Invest.,* **82,** 25–35.

Iglesias-de la Cruz, M. C., Ziyadeh, F. N., Isono, M., Kouahou, M., Han, D. C., Kalluri, R., Mundel, P., and Chen, S. (2002) Effects of high glucose and TGF-betal on the expression of collagen IV and vascular endothelial growth factor in mouse podocytes. *Kidney Int.,* **62,** 901–913.

Inoki, K., Haneda, M., Maeda, S., Koya, D., and Kikkawa, R. (1999) TGF-beta 1 stimulates glucose uptake by enhancing GLUT1 expression in mesangial cells. *Kidney Int.,* **55,** 1704–1712.

Kaiser, N., Sasson, S., Feener, E. P., Boukobza-Vardi, N., Higashi, S., Moller, D. E., Davidheiser, S., Przybylski, R. J., and King, G. L. (1993) Differential regulation of glucose transport and transporters by glucose in vascular endothelial and smooth muscle cells. *Diabetes,* **42,** 80–89.

Kelly, D. J., Gilbert, R. E., Cox, A. J., Soulis, T., Jerums, G., and Cooper, M. E. (2001) Aminoguanidine ameliorates overexpression of prosclerotic growth factors and collagen deposition in experimental diabetic nephropathy. *J. Am. Soc. Nephrol.,* **10,** 2098–2107.

Kumagai, A. K., Vinores, S. A., and Pardridge, W. M. (1996) Pathological upregulation of inner blood-retinal barrier Glut1 glucose transporter expression in diabetes mellitus. *Brain Res.,* **706,** 313–317.

Landau, D., Segev, Y., Afargan, M., Silbergeld, A., Katchko, L., Podshyvalov, A., and Phillip, M. (2001) A novel somatostatin analogue prevents early renal complications in the nonobese diabetic mouse. *Kidney Int.,* **60,** 505–512.

Liu, Z. H., Chen, Z. H., Li, Y.-J., Liu, D., and Li, L. S. (1999) Phenotypic alterations of live mesangial cells in patients with diabetic nephropathy obtained from renal biopsy specimen [abstract]. *J. Am. Soc. Nephrol.,* **10,** A0669.

Lupia, E., Elliot, S. J., Lenz, O., Zheng, F., Hattori, M., Striker, G. E., and Striker, L. J. (1999) IGF-1 decreases collagen degradation in diabetic NOD mesangial cells: Implications for diabetic nephropathy. *Diabetes,* **48,** 1638–1644.

MacKay, K., Striker, L. J., Stauffer, J. W., Doi, T., Agodoa, L. Y., and Striker, G. E. (1989) Transforming growth factor-beta. Murine glomerular receptors and responses of isolated glomerular cells. *J. Clin. Invest.,* **83,** 1160–1167.

Mauer, S. M., Steffes, M. W., Ellis, E. N., Sutherland, D. E., Brown, D. M., and Goetz, F. C. J. (1984) Structural-functional relationships in diabetic nephropathy. *J. Clin. Invest.,* **74,** 1143–1455.

Meyer, T. W., Bennett, P. H., and Nelson, R. G. (1999) Podocyte number predicts long-term urinary albumin excretion in Pima Indians with type II diabetes and mircoalbuminuria. *Diabetologia,* **42,** 1341–1344.

O'Donnell, M. P., Burne, M., Daniels, F., and Rabb, H. (2002) Utility and limitations of serum creatinine as a measure of renal function in experimental renal ischemia-reperfusion injury. *Transplantation,* **73,** 1841–1844.

Pagtalunan, M. E., Miller, P. L., Jumping-Eagle, S., Nelson, R. G., Myers, B. D., Rennke, H. G., Coplon, N. S., Sun, L., and

Meyer, T. W. (1997) Podocyte loss and progressive glomerular injury in type II diabetes. *J. Clin. Invest.,* **99,** 342–348.

Riley, S. G., Steadman, R., Williams, J. D., Floege, J., and Phillips, A. O. (1999) Augmentation of kidney injury by basic fibroblast growth factor or platelet-derived growth factor does not induce progressive diabetic nephropathy in the Goto Kakizaki model of non-insulin-dependent diabetes. *J. Lab. Clin. Med.,* **134,** 304–312.

Riser, B. L., Denichilo, M., Cortes, P., Baker, C., Grondin, J. M., Yee, J., and Narins, R. G. (2000) Regulation of connective tissue growth factor activity in cultured rat mesangial cells and its expression in experimental diabetic glomerulosclerosis. *J. Am. Soc. Nephrol.,* **11,** 25–38.

Schiffer, M., Bitzer, M., Roberts, I. S., Kopp, J. B., ten Dijke, P., Mundel, P., and Bottinger, E. P. (2001) Apoptosis in podocytes induced by TGF-beta and Smad7. *J. Clin. Invest.,* **108,** 807–816.

Schiffer, M., Schiffer, L. E., Gupta, A., Shaw, A. S., Roberts, I. S., Mundel, P., and Bottinger, E. P. (2002) Inhibitory Smads and TGF-beta signaling in glomerular cells. *J. Am. Soc. Nephrol.,* **13,** 2657–2666.

Shankland, S. J., and Scholey, J. W. (1994) Expression of transforming growth factor $\beta 1$ during diabetic renal hypertrophy. *Kidney Int.,* **46,** 430–442.

Sharma, K., and Ziyadeh, F. N. (1994) Renal hypertrophy is associated with upregulation of TGF-$\beta 1$ gene expression in diabetic BB rat and NOD mouse. *Am. J. Physiol.,* **67,** F1094–F1101.

Shimada, K., Baba, T., Neugebauer, S., Onozaki, A., Yamada, D., Midorikawa, S., Sato, W., and Watanabe, T. (2002) Plasma vascular endothelial growth factor in Japanese type 2 diabetic patients with and without nephropathy. *J. Diabetes Complications,* **16,** 386–390.

Sugimoto, H., Hamano, Y., Charytan, D., Cosgrove, D., Kieran, M., Sudhakar, A., and Kalluri, R. (2003) Neutralization of circulating vascular endothelial growth factor (VEGF) by anti-VEGF antibodies and soluble VEGF receptor 1 (sFlt-1) induces proteinuria. *J. Biol. Chem.,* **278,** 12605–12608.

Thrailkill, K. M. (2000) Insulin-like growth factor-I in diabetes mellitus: Its physiology, metabolic effects, and potential clinical utility. *Diabetes Technol. Ther.,* **2,** 69–80.

Twigg, S. M., Cao, Z., McLennan, S. V., Burns, W. C., Brammar, G., Forbes, J. M., and Cooper, M. E. (2002) Renal connective tissue growth factor induction in experimental diabetes is prevented by aminoguanidine. *Endocrinology,* **143,** 4907–4915.

Twigg, S. M., Chen, M. M., Joly, A. H., Chakrapani, S. D., Tsubaki, J., Kim, H. S., Oh, Y., and Rosenfeld, R. G. (2001) Advanced glycosylation end products up-regulate connective tissue growth factor (insulin-like growth factor-binding protein-related protein 2) in human fibroblasts: A potential mechanism for expansion of extracellular matrix in diabetes mellitus. *Endocrinology,* **142,** 1760–1769.

U.S. Renal Data System (USRDS). (2002) *Annual Data Report: Atlas of End-Stage Renal Disease in the United States* (2002). Bethesda, MD, National Institutes of Health, National Institute of Diabetes and Digestive and Kidney Diseases.

Vlassara, H., and Palace, M. R. (2002) Diabetes and advanced glycation endproducts. *J. Intern. Med.,* **251,** 87–101.

Wahab, N. A., Yevdokimova, N., Weston, B. S., Roberts, T., Li, X. J., Brinkman, H., and Mason, R. M. (2001) Role of connective tissue growth factor in the pathogenesis of diabetic nephropathy. *Biochem. J.,* **359,** 77–87.

Wang, S. N., Lapage, J., and Hirschberg, R. (2001) Loss of tubular bone morphogenetic protein-7 in diabetic nephropathy. *J. Am. Soc. Nephrol.,* **12,** 2392–2399.

Weigert, C., Brodbeck, K., Brosius, F. C., III, Huber, M., Lehmann, R., Friess, U., Aulwurm, S., Häring, H., Schleicher, E. D., and Heilig, C. W. (2003) Evidence for a novel TGF-$\beta 1$-independent mechanism of fibrosis in mesangial cells overexpressing glucose transporters. *Diabetes,* **52,** 527–535.

Weston, B. S., Wahab, N. A., and Mason, R. M. (2003) CTGF mediates TGF-beta-induced fibronectin matrix deposition by upregulating active alpha5beta1 integrin in human mesangial cells. *J. Am. Soc. Nephrol.,* **14,** 601–610.

Yamagishi, S., Inagaki, Y., Okamoto, T., Amano, S., Koga, K., Takeuchi, M., and Makita, Z. (2002) Advanced glycation end product-induced apoptosis and overexpression of vascular endothelial growth factor and monocyte chemoattractant protein-1 in human-cultured mesangial cells. *J. Biol. Chem.,* **277,** 20309–20315.

Ziyadeh, F. N., Sharma, K., Ericksen, M., and Wolf, G. (1994) Stimulation of collagen gene expression and protein synthesis in murine mesangial cells by high glucose is mediated by autocrine activation of transforming growth factor-beta. *J. Clin. Invest.,* **93,** 536–542.

Ziyadeh, F. N., Hoffman, B. B., Han, D. C., Iglesias-De La Cruz, M. C., Hong, S. W., Isono, M., Chen, S., McGowan, T. A., and Sharma, K. (2000) Long-term prevention of renal insufficiency, excess matrix gene expression, and glomerular mesangial matrix expansion by treatment with monoclonal antitransforming growth factor-beta antibody in db/db diabetic mice. *Proc. Natl. Acad. Sci. U.S.A.,* **97,** 8015–8020.

